# Study of the *Bacillus thuringiensis* Cry1Ia Protein Oligomerization Promoted by Midgut Brush Border Membrane Vesicles of Lepidopteran and Coleopteran Insects, or Cultured Insect Cells

**DOI:** 10.3390/toxins12020133

**Published:** 2020-02-21

**Authors:** Ayda Khorramnejad, Mikel Domínguez-Arrizabalaga, Primitivo Caballero, Baltasar Escriche, Yolanda Bel

**Affiliations:** 1 Departamento de Genética/ERI BioTecMed, Universitat de València, Burjassot, 46100 València, Spain; ayda.khoramnejad@uv.es (A.K.); baltasar.escriche@uv.es (B.E.); 2 Department of Plant Protection, College of Agriculture and Natural Resources, University of Tehran, Karaj 31578-77871, Alborz, Iran; 3 Departamento de Agronomía, Biotecnología y Alimentación, Universidad Pública de Navarra, Pamplona, 31006 Navarra, Spain; mikel.dominguez@unavarra.es (M.D.-A.); pcm92@unavarra.es (P.C.)

**Keywords:** Cry1Ab, oligomer formation, Sf21 cell line, *Ostrinia nubilalis*, *Lobesia botrana*, *Leptinotarsa decemlineata*, bioassay

## Abstract

*Bacillus thuringiensis* (Bt) produces insecticidal proteins that are either secreted during the vegetative growth phase or accumulated in the crystal inclusions (Cry proteins) in the stationary phase. Cry1I proteins share the three domain (3D) structure typical of crystal proteins but are secreted to the media early in the stationary growth phase. In the generally accepted mode of action of 3D Cry proteins (sequential binding model), the formation of an oligomer (tetramer) has been described as a major step, necessary for pore formation and subsequent toxicity. To know if this could be extended to Cry1I proteins, the formation of Cry1Ia oligomers was studied by Western blot, after the incubation of trypsin activated Cry1Ia with insect brush border membrane vesicles (BBMV) or insect cultured cells, using Cry1Ab as control. Our results showed that Cry1Ia oligomers were observed only after incubation with susceptible coleopteran BBMV, but not following incubation with susceptible lepidopteran BBMV or non-susceptible Sf21 insect cells, while Cry1Ab oligomers were persistently detected after incubation with all insect tissues tested, regardless of its host susceptibility. The data suggested oligomerization may not necessarily be a requirement for the toxicity of Cry1I proteins.

## 1. Introduction

The entomopathogenic Gram-positive bacterium, *Bacillus thuringiensis* (Bt), is the most successful bioinsecticide commercialized to date. It generates a wide variety of insecticidal proteins that can be produced at different growth stages. Cry, Cyt and parasporin proteins are synthetized during the stationary growth phase, Cry1I proteins are secreted in the initial phase of sporulation and Vip and Sip proteins are secreted during the vegetative phase of bacterial growth [1,2,3,4,5]. The success of Bt-based insecticides is due to its narrow spectrum of activity, environmental safety and because it is harmless to animals and plants [6]. To date, the most studied Bt entomopathogenic proteins are the three domain (3D) Cry proteins such as Cry1 and Cry2 toxins. Their mode of action is not completely known, but it is commonly accepted that specific binding to insect midgut receptors is essential to exert their toxicity [7,8]. The current models of Cry toxin action include the “signal transduction model” that claims that the toxicity is mediated by intracellular pathways [9], and the “sequential mode of action”—the most accepted model so far, that is based on the sequential binding of Bt toxin to several midgut receptors, the promotion of a pre-pore oligomer, the insertion of pre-pore oligomer into the midgut membrane, pore formation, osmotic imbalance, midgut epithelium disruption, septicemia and insect death [10]. Regarding this model, some authors have stated that the formation of the oligomer prior to toxin insertion into membrane is a major step in the toxicity process, and that the oligomeric (tetrameric) structure, is necessary for the final pore formation; also, it has been claimed that the oligomer formation is a conserved mechanism in the mode of action of the Cry proteins [11,12,13,14,15,16].

Oligomerization has been studied in several wild type and mutant toxins such as Cry1Aa, Cry1Ab, Cry1Ac, Cry1Ca, Cry1Da, Cry1Ea, Cry1Fa, Cry2Ab, Cry3Aa, Cry3Ba, Cry3Ca, Cry4Ba, Cry11Aa and Cry46Aa1 [15,17,18,19,20,21,22,23,24,25]. Oligomerization has also been detected in other Bt toxins such as Cyt [26] and Vip [27]. However, regarding the Cry1I protein family, only one study has described the possible oligomerization of this protein in solution, in the absence of insect midgut proteins [28]. So far, the promotion of Cry1I oligomer formation after incubation with insect tissues or insect cell-derived models has never been experimentally addressed.

Cry1I proteins are included within the Cry1 family (3D Cry proteins) because of their sequence similarities and structural characteristics. However, Cry1I proteins display several unique, specific and remarkable characteristics, that include their secretion early in the stationary growth phase of Bt (instead of forming part of the Bt crystals), their unusual protoxin molecular weight (80 kDa), and the dual insecticidal activity against lepidopteran and coleopteran pests [29,30]. This, together with the lack of cross-resistance with other Cry1A and Cry1F insecticidal proteins [31,32,33], make Cry1I proteins interesting for developing new insecticidal products and indeed, they have been recently introduced in Bt crops [34,35].

Understanding the mode of action of Bt toxins is critical for enhancing and sustaining their efficacy against pests. In this context, in the present work, for the first time, the oligomer formation of Cry1Ia toxins has been examined after incubation with midgut insect tissues, trying to clarify its relevance in the Cry1I mode of action. Cry1Ab, for which oligomerization has been reported in several studies [36,37,38,39], has been used as control. In this study, midgut brush border membrane vesicles (BBMV) of different insect species from Lepidoptera (*Ostrinia nubilalis* and *Lobesia botrana*) and Coleoptera (*Leptinotarsa decemlineata*), as well as a cell line derived from *Spodoptera frugiperda* (Sf21) were used to promote the Cry oligomer formation. This selection covered both susceptible and tolerant insect species for Cry1Ia and Cry1Ab. Based on this, the oligomer formation of Cry1Ia toxin compared to Cry1Ab, and its possible correlation with insecticidal activity, have been examined.

## 2. Results

### 2.1. Toxicity of Cry1Ia and Cry1Ab against Lepidopteran and Coleopteran Species

The toxicity of Cry1Ia and Cry1Ab protoxins towards the insects’ species used in the present study were assessed by performing bioassays. The results are summarized in Table 1. The Cry1Ia protein was found to be toxic for the two selected lepidopteran species, *L. botrana*, and *O. nubilalis*, with LC_50_ values of 80 and 273 ng/cm^2^ respectively, as well as for the coleopteran *L. decemlineata* (LC_50_ = 22 µg/mL). On the other hand, Cry1Ab was only toxic for the lepidopteran insect species.

### 2.2. BBMV of Susceptible Lepidopteran Insects Promoted Oligomerization of Cry1Ab But Not of Cry1Ia

**Figure 1:**
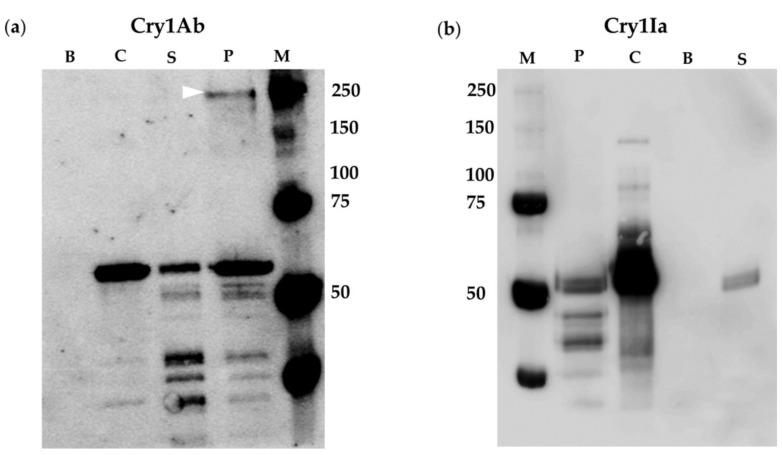
Cry1Ab and Cry1Ia oligomer formation promoted by *O. nubilalis* BBMV: (**a**) Cry1Ab; (**b**) Cry1Ia. Lanes B: *O. nubilalis* BBMV incubated without Cry1Ab or Cry1Ia proteins. Lanes C: Control of Cry1Ab or Cry1Ia proteins, incubated without BBMV. Lanes S: Supernatant obtained after incubation of Cry1Ab or Cry1Ia proteins, with the BBMV. Lanes P: Pellet obtained after incubation of Cry1Ab or Cry1Ia with the BBMV. Lanes M: Molecular weight marker. The arrowhead points to the Cry1Ab oligomer (about 250 kDa).

The oligomer formation of Cry1Ab and Cry1Ia was studied by incubating the proteins with lepidopteran BBMV from *O. nubilalis* and *L. botrana*, susceptible hosts (Table 1). In order to favor oligomer detection, the milder SDS-PAGE denaturing conditions used in the bibliography to observe Cry1 oligomers were employed (see Section 4.8 in Materials and Methods). The results showed that Cry1Ab toxin was able to form oligomers after incubation with BBMV from *O. nubilalis*, but this incubation did not promote Cry1Ia oligomer formation (Figure 1). The oligomers observed (band of about 250 kDa) were associated to the *O. nubilalis* BBMV fraction (Figure 1a, lane P); these oligomeric structures could be inserted into the membranes or just bound to the surface of the BBMV due to interaction with specific membrane proteins. For Cry1Ab, the Western blot revealed another band with a molecular weight of approximately 60 kDa, corresponding to the monomeric form of Cry1Ab (Figure 1a, lane P). Nevertheless, the Cry1Ia protein associated with *O. nubilalis* BBMV was detected as a single band of about 50 kDa, corresponding to Cry1Ia monomers, and no band corresponding to Cry1Ia oligomers was detected (Figure 1b, lane P). The Cry1Ab and Cry1Ia monomeric proteins were also recovered in the supernatants, as bands of about 60 and 50 kDa, respectively (Figure 1, lanes S). Controls of Cry1Ab or Cry1Ia proteins without BBMV, but subjected to the same process as the rest of the samples, were conducted to assess the possible spontaneous formation of oligomers. The results (Figure 1, lanes C) showed the presence of Cry1Ab and Cry1Ia monomers mostly while the oligomers (tetramers) were not detected in these lanes, pointing to the fact that Cry1Ab and Cry1Ia tetramers did not form spontaneously in the solution in the absence of insect BBMV. In the case of Cry1Ia some minor bands of MW about 65 and 90 kDa were detected, which were most probably traces of Cry1Ia protoxin and partially trypsinized products; a third minor band of about 130 kDa (MW that does not match with the size of Cry1Ia dimers or trimers) was also observed.

**Figure 2:**
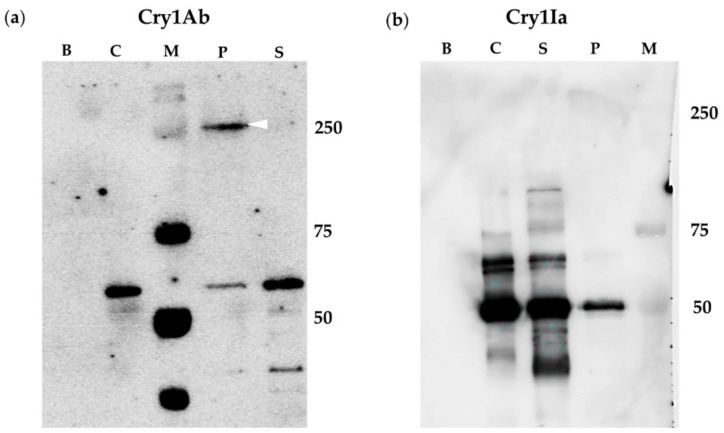
Cry1Ab and Cry1Ia oligomer formation promoted by *L. botrana* BBMV: (**a**) Cry1Ab; (**b**) Cry1Ia. Lanes B: *L. botrana* BBMV incubated without Cry1Ab or Cry1Ia proteins. Lanes C: Controls of Cry1Ab or Cry1Ia proteins incubated without BBMV. Lanes P: Pellet obtained after incubation of the respective protein with the *L. botrana* BBMV. Lanes S: Supernatant obtained after incubation of the respective protein with the *L. botrana* BBMV. Lanes M: molecular weight marker. The arrowhead points to the Cry1Ab oligomer (about 250 kDa).

The oligomer formation was also tested using BBMV from *L. botrana*. The results obtained were similar to the ones found with *O. nubilalis* BBMV. The incubation of Cry1Ia with BBMV from *L. botrana*, did not render bands with molecular weight consistent with Cry1Ia oligomeric structures (Figure 2b, lane P), whilst, after incubation of Cry1Ab with *L. botrana* BBMV, a clear band corresponding to a Cry1Ab oligomer (tetramer) was observed (Figure 2, lane P). On the other hand, monomers of both proteins were detected in the corresponding supernatants recovered after the BBMV-protein incubation assays (Figure 2, lanes S). In the Cry1Ia experiments, minor bands of MW of about 65 and 90 kDa were observed in the supernatant (S) and control (C) lanes that probably represent a minor fraction of partially trypsinized Cry1Ia.

### 2.3. Oligomerization of Cry1Ia Was Promoted by BBMV from L. decemlineata

Cry1Ia exhibits a dual toxic activity towards lepidopteran and coleopteran hosts, whereas Cry1Ab is only toxic to lepidopteran species. In this work, *L. decemlineata* BBMV was employed as a coleopteran Cry1Ia susceptible host tissue (Table 1) to study Cry1Ia oligomer promotion as well to study if these BBMV promoted the oligomerization of the Cry1Ab protein, non-toxic for this insect species (Table 1).

**Figure 3:**
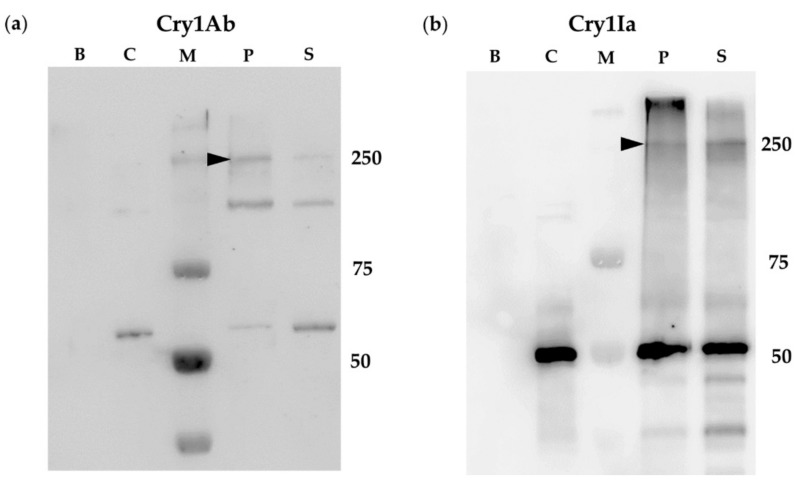
Cry1Ab and Cry1Ia oligomer formation promoted by *L. decemlineata* BBMV: (**a**) Cry1Ab; (**b**) Cry1Ia. Lanes B: *L. decemlineata* BBMV incubated without Cry1Ab or Cry1Ia proteins. Lanes C: Controls of Cry1Ab or Cry1Ia proteins incubated without BBMV. Lanes P: Pellet obtained after incubation of the respective protein with the *L. decemlineata* BBMV. Lanes S: Supernatant obtained after incubation of the respective protein with the *L. decemlineata* BBMV. Lanes M: molecular weight marker. The arrowheads in panels (**a**,**b**) point to the Cry1Ab (about 250 kDa) and the Cry1Ia (about 250 kDa) oligomer bands respectively.

The results, summarized in Figure 3, showed that the incubation of either Cry1Ab or Cry1Ia toxins with the coleopteran BBMV provided bands of a molecular weight of about 250 kDa for both Cry proteins, corresponding to the respective oligomers. Interestingly, the oligomers were detected in both, pelleted (BBMV) and supernatant fractions (Figure 3, lanes P and S respectively). Bands corresponding to the monomers of both proteins were also observed in both fractions (pelleted BBMV and supernatant) for both Cry proteins. Incubation with Cry1Ab rendered also a band of about 150 kDa that could correspond to a dimer. It is worth highlighting that the band corresponding to the Cry1Ia oligomer had a molecular weight of about 250 kDa, which would indicate that Cry1Ia oligomer could be composed by more than four subunits, since the Cry1Ia monomer has a size of about 50 kDa. Moreover, a strong band of molecular weight higher than 250 kDa was also observed as being associated to the BBMV (Figure 3, lane P) that could correspond to Cry1Ia aggregates with high number of units.

### 2.4. Oligomerization Promoted by Sf21 Insect Cells

**Figure 4:**
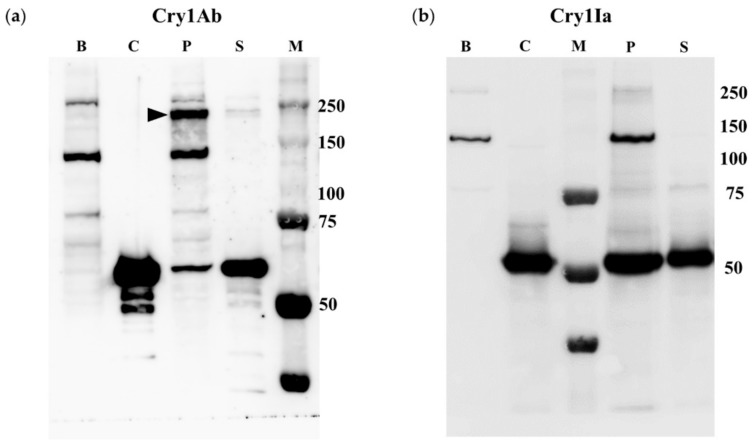
Cry1Ab and Cry1Ia oligomer formation promoted by Sf21 cells: (**a**) Cry1Ab; (**b**) Cry1Ia. Lanes B: Sf21 cells incubated without Cry1Ab or Cry1Ia proteins. Lanes C: Controls of Cry1Ab or Cry1Ia proteins incubated without Sf21 cells. Lanes P: Pellet obtained after incubation of the respective protein with the Sf21 cells. Lanes S: Supernatant obtained after incubation of the respective protein with the Sf21cells. Lanes M: molecular weight marker. The arrowhead in Cry1Ab panel points to the Cry1Ab oligomer.

It has been described that Sf21 cells (insect cultured cells derived from *S. frugiperda* ovaries) are tolerant to both Cry1Ab and Cry1Ia proteins [40,41]. These insect cells were selected in order to check if oligomer formation could be promoted by tolerant insect tissues. Figure 4 shows the results of the incubation of Sf21 cells with each one of these proteins. In the case of Cry1Ab, the incubation of the protein with the insect cells resulted in the detection of two main bands of approximately 60 and 250 kDa associated with the cell fraction, corresponding to Cry1Ab monomers and oligomers, respectively (Figure 4a, lane P). Other minor bands detected were also present in the control lanes of Sf21 cells that had been incubated without Cry proteins, showing that these bands probably correspond to natural biotinylated proteins present in the cells (Figure 4, lanes B). In the case of Sf21 cells incubated with Cry1Ia, only a band with the molecular weight of the monomer (50 kDa) was found to be associated with the cells (Figure 4b, lane P). In summary, results pointed out the absence of Cry1Ia oligomer formation after incubation with Sf21 cells, in contrast to what is observed after incubation with Cry1Ab. It is worth noting that in the supernatants, only a main band with the molecular weight of the Cry1Ab or Cry1Ia monomers, was detected (Figure 4, lanes S).

## 3. Discussion

Cry1I proteins share sequence and structural similarities to the most-known three domain Cry proteins present in the parasporal crystal of Bt [10,29,42]. Therefore, so far, it has been assumed that their mode of action is similar to its crystal protein counterparts, despite their special features such as that the Cry1I proteins do not form crystals [5,43], their protoxin MW is smaller (about 81 kDa [29]), and they show dual toxic activity against lepidopteran and coleopteran insect pests [29,44].

The mode of action of the Cry proteins accumulated in the crystals is not completely understood [10,12]. It is commonly accepted that includes the solubilization of the crystals in the insect midgut to yield the protoxin form, and a proteolytic processing to produce the activated forms. Then, the “sequential binding model”, suggests that the activated proteins bind to several midgut membrane receptors to finally form an oligomeric structure that inserts in the midgut membrane and forms pores, leading to cellular osmotic imbalance, cell lysis, septicaemia and eventually insect death [13,14]. In support of this model, several studies have reported that oligomerization plays a crucial role in the insecticidal activity of *B. thuringiensis* Cry toxins [18,38,45,46]. Moreover, Cry protein mutants that did not form oligomers, showed severely decreased toxicity [17,24,45]. Likewise, it has been shown that some Cry1A mutants that had lost their toxicity, were unable to oligomerize and to form pores [38,47]. However, despite these studies, the sequential binding to several insect membrane proteins as well as the oligomer formation and insertion events prior to pore formation are not clearly defined yet [12]. On the other hand, as an alternative to the “sequential binding model”, the “signaling pathway model” has been proposed. This model claims that the Cry toxicity can be due to the activation of intracellular cell death pathways [9]. Indeed, some authors claim that both mechanisms could coexist [48].

In this context, the occurrence of an oligomerization step in the mode of action of Cry1Ia protein, and the study of Cry1Ia oligomerization promotion by membranes of susceptible and tolerant insect BBMV or insect cells, have been the main goals of this research.

In 2009, the occurrence of a spontaneous oligomerization of trypsinized Cry1Ie in solution was reported [28]. According to the mentioned research, the oligomer fraction contained a small amount of dimer and a large amount of aggregates larger than tetramers. The toxicity of the oligomers against *Plutella xylostella* was about 70 times lower than the toxicity of the monomer or the toxicity of the non-trypsinized Cry1Ie protein, which led the authors to claim that the Cry1Ie spontaneous aggregation most likely differ from the oligomers occur by the insect midgut membranes. In the present work, the oligomerization of Cry1Ia protein after incubation with susceptible insect BBMV or with non-susceptible cultured insect cells, has been determined for the first time. 

To properly select the insect species for this study, the insecticidal activity of Cry1Ia and Cry1Ab protoxins towards two lepidopteran species (*O. nubilalis* and *L. botrana*) and a coleopteran insect (*L. decemlineta*) has been assessed. The toxicity data obtained were expected based on the published data [29,44,49,50]. Moreover, Sf21 cells were also used in this study, on the bases that Cry1Ia and Cry1Ab are not toxic for them [40,41].

So far, Cry oligomerization studies have focused on the incubation of the Cry proteins with BBMV of susceptible and resistant populations of the same insect, and, as a result, an association has been found between resistance and reduced oligomerization [51,52]. In the present work, we have used Cry1Ab as an oligomeric control protein [36,37,38,39], and have been able to clearly detect Cry1Ab oligomers in the expected tetramer form (about 250 kDa molecular weight size) after incubation with all tested insect tissues, regardless to their susceptibility. Thus, oligomers have been observed after incubation of the trypsinized Cry1Ab protein with *O. nubilalis* or *L. botrana* BBMV (susceptible insects), but also with *L. decemlineata* BBMV (non-susceptible insect) and Sf21 cells (non-susceptible cultured insect cells). The oligomeric structures were found associated to the insect BBMV or to the insect cells, indicating that they were either inserted or bound to the membrane proteins. The finding of Cry1Ab oligomers after incubation with tolerant insect BBMV or associated with tolerant insect cells could be explained by an improper insertion of oligomers into the membranes (and therefore inefficiency to produce damages), or by the inability to induce the post-pore subsequent events in the cells (e.g., no triggering of cell death mechanisms).

In this study, the Cry1Ab oligomer was mainly found to be associated to the membrane fractions in accordance with previous reports, but oligomers were also observed in the supernatants after incubation with BBMV from the non-susceptible insect *L. decemlineata*. This suggested that the Cry1Ab oligomers (tetramers and dimers), promoted by the coleopteran BBMV, are not only associated to the BBMV (whether inserted or not) but also free in the supernatant (Figure 3a, lane S). The results obtained resemble the ones shown by Rodríguez-Almazán et al. [47], with the Cry1Ab helix α-4 mutants which had a mutation in domain I, involved in membrane insertion and pore formation, and had lost drastically their insecticidal activity towards *M. sexta* larvae. Similarly, in our results, the incubation of Cry1Ab with BBMV from *L. decemlineata* rendered a relatively high amount of oligomeric structures (dimers and tetramers) in the supernatant, apparently indicating that these oligomers were not able to insert into the membranes, resulting in the absence of toxicity. Nevertheless, in this study, after the incubation of the Cry1Ia protein with *L. decemlineata* BBMV, a high proportion of Cry1Ia oligomers were also observed in the supernatants (Figure 3b, laneS), and in this case, the protein showed a high toxicity against this coleopteran pest (Table 1).

The Cry1Ia oligomers were observed after incubation of the protein with BBMV from the susceptible coleopteran *L. decemlineata*. The molecular weights of the bands that correspond to the Cry1Ia oligomers (about 250 kDa) point out that the Cry1Ia oligomer could be composed of more than four units. 

After incubation of Cry1Ia with BBMV of susceptible lepidopterans (*O. nubilalis* and *L. botrana*) or with insect Sf21 cells, no oligomers were detected, suggesting that the Cry1Ia proteins associated to the lepidopteran membranes could be mainly in monomeric forms. In this case, oligomers would not be a limiting step in the toxicity of Cry1Ia proteins, and the toxicity could be mediated by intracellular signaling pathways [9]. Moreover, other hypotheses could be mentioned to explain the reason for the absence of Cry1Ia oligomers. Firstly, it can be suggested that the observed monomers come from disassembled oligomers produced due to the SDS-PAGE technique conditions, similarly to what was proposed by Ocelotl et al. [51] working with Cry1Ab oligomers. However, this reasoning would be in conflict with the observation of oligomers after the incubation of Cry1I with coleopteran BBMV, which were detected using the same SDS-PAGE conditions. Secondly, it could be considered that the biotinlylation of Cry1I could interfere with oligomerization. This hypothesis was examined following the incubation of the biotin labelled Cry1Ab with *O. nubilalis* BBMV. The results showed no influence of biotin in oligomerization (Figure S1), similarly to what had been already claimed by other authors showing that biotinylation of Cry proteins does not prevent their oligomerization, binding and insecticidal activity [12,53,54]. Thirdly, it has to be noticed that, in the present study, the oligomer formation experiments were performed with trypsinized Cry1Ab and Cry1Ia proteins to mimic the in vivo conditions. It has been described that Cry1Ie protoxin and trypsinized protein have the same toxicity [28]. However, other Cry1I proteins such as Cry1Ia1 have shown some differences in toxicity amongst protoxins (more active) and trypsinized proteins [49]. We can speculate that in vitro trypsinization of Cry1Ia could alter the toxicity by impairing oligomer formation, maybe provoking a flawed ability to form oligomers when they are promoted by lepidopteran BBMV.

The spontaneous formation of Cry1Ab and Cry1Ia oligomers in solution has been questioned in this study. Cry1Ab submitted to the same experimental situation of treatments, but without being in contact with insect BBMV or cells, did not oligomerize (Figure 1, Figure 2, Figure 3 and Figure 4a, lanes C). Regarding Cry1Ia, some bands of smaller sizes than the expected tetramer, and with MW sizes that were not multiples of 50 kDa (monomer size) were observed (Figure 1, Figure 2, Figure 3 and Figure 4b, lanes C). Most probably, these bands are residual incomplete trypsinized Cry1Ia forms, dragged through the toxin purification process. In conclusion, although some studies have reported the spontaneous formation of Cry protein oligomers in solution without being in contact with the membrane-like environment (i.e., Cry4Ba [55] or Cry2Ab [24]), in our study neither Cry1Ab nor Cry1Ia formed oligomers without being exposed to insect BBMV or cultured insect cells. 

In summary, our findings indicated that the oligomers of a classical 3D crystal forming Cry protein of Bt such as Cry1Ab were promoted and could be detected after incubation of activated Cry1Ab with susceptible or non-susceptible insect midgut BBMV and with non- susceptible Sf21 cells. In contrast, in the same assay conditions, Cry1Ia oligomers were detected only after incubation with BBMV of *L. decemlineata* (coleopteran susceptible host), but no Cry1Ia oligomeric structures were found following incubation of Cry1Ia with lepidopteran BBMV or with the Sf21 cells, regardless of its host susceptibility. Hence, our results, using trypsin processed Cry1Ab and Cry1Ia as an in vitro model of what might occur in vivo, suggest that: (1) The promotion of oligomers can occur by incubation of the Cry toxin with susceptible insect BBMV but also with non-susceptible insect tissues, and (2) The oligomerization may not be a determining step in the toxicity of Cry1I proteins.

## 4. Materials and Methods

### 4.1. Production and Purification of Cry Proteins

The Cry1Ab protein used for oligomer formation was obtained from a recombinant *Escherichia coli* strain GG094-208 (kindly supplied by Dr. R.A. de Maagd, Wageningen University, The Netherlands). Protein expression, inclusion bodies purification, solubilization and protoxin activation by trypsin, were performed as described previously [56]. The activated Cry1Ab was purified by anion-exchange chromatography using Äkta 100 explorer system (GE Healthcare, Amersham, UK) following Crava et al. [57]. The eluted fractions from the column were individually analysed by sodium dodecyl sulphate-12% polyacrylamide gel electrophoresis (SDS-PAGE).

The *cry1Ia7* gene was cloned and expressed in *E. coli*, BL21(DE3) cells [40]. The purification of the protein by affinity chromatography using a HisTrapTM FF crude column (GE Healthcare Bio-Sciences, Upsala, Sweden), protein dialysis and protoxin activation by trypsin, were performed as reported by Khorramnejad et al. [40]. The activated Cry1Ab and Cry1Ia were visualized after SDS-PAGE, and their concentration was estimated by densitometry using TotalLab Quant program version 12.3 (Newcastle, UK), employing bovine serum albumin as standard. 

The Cry1Ab protein used in bioassays (protoxin) was obtained from a recombinant Bt strain that produced a crystal composed solely of the Cry1Ab protein, kindly supplied by Dr. Colin Berry, Cardiff University, Cardiff, UK. This Bt strain was grown in CCY medium [58] supplemented with erythromycin and the crystal formation was observed daily. When at least 95% of the cells were lysed, the fermentation process was stopped and then spores and crystals were collected by centrifugation (8600× *g*, 4 °C), washed with a saline solution (NaCl 1M, EDTA 10mM) and resuspended in KCl (10 mM). For Cry1Ia7 protoxin production, the recombinant *E. coli* BL21 (DE3) was grown and purified as described above. Both Cry1Ab and Cry1Ia7 protoxins were quantified by the method of Bradford [59] using BSA as standard, and kept at 4 °C until used.

### 4.2. Insect Rearing

Two different lepidopteran species, *L. botrana* (Lep: Tortricide) and *O. nubilalis* Hübner (Lep.: Crambidae), and one coleopteran species, *L. decemlineata* (Col.: Chrysomelidae) (the Colorado potato beetle, CPB) were used in this study. Both lepidopteran pests were maintained in the insectary of the Universidad Pública de Navarra (UPNA, Pamplona, Spain) at 25 °C ± 1 °C with 70% ± 5% RH and a photoperiod of 16/8 h (light/dark) on the artificial diet described by MacIntosh et al. [60]. The population of beetles was raised on potato plants (Desiré variety) grown throughout the year in a phytotron to provide a continuous supply of food substrate. This population was refreshed 1–2 times every year by the introduction of wild adults collected in the field during the spring-summer.

### 4.3. Insect Cell Line

The lepidopteran cell line Sf21, from ovaries of fall armyworm *S. frugiperda* (Lep.: Noctuidea), was obtained from Wageningen University (Wageningen, The Netherlands). The Sf21 cells were maintained at 25 °C on 1X Grace’s medium (Gibco^®^ Life technologies^TM^, Carslbad, CA, USA) supplemented with 10% heat-inactivated fetal bovine serum (FBS) in 25 cm^2^ cell culture flasks. Cells were passaged weekly. Cell concentrations were measured by using an automatic cell counter (Countess Automated Cell Counter from Invitrogen, Carlsbad, CA, USA).

### 4.4. Insect Bioassays

The bioassays for the three insect species were performed with first instar larvae. Five different protoxin concentrations, ranging from 0.39 to 100 μg/mL, were prepared to determine the concentration-mortality responses in order to calculate the mean lethal concentration (LC_50_). For the lepidopteran insect species, *O. nubilalis* and *L. botrana*, the diet surface contamination assay was used [61], and for *L. decemlineata*, the leaf dip bioassay described by Iriarte et al. [62], was performed. For each bioassay, several protein concentrations were tested, using 28 larvae per concentration. Each bioassay was repeated at least three times. Total insect mortality was recorded after 7 days for lepidopteran insects, and after 4 days for *L. decemlineata*. The concentration-mortality data obtained for each insect species were analyzed after transformation of the concentration-response curve to fit a linear model using POLO-PC program (LeOra Software, Berkeley, CA, USA, 1987), based on the Probit analysis [63].

### 4.5. Midgut Isolation and BBMV Preparation

Midguts were dissected from fifth-instar larvae of *O. nubilalis* and forth-instar larvae of *L. decemlineata*. The dissected midguts were rinsed in ice-cold MET buffer (0.3 M mannitol, 5 mM EGTA, 17 mM Trsi-HCl, pH 7.5), snap frozen in liquid nitrogen and kept at −80 °C until use.

Brush border membrane vesicles (BBMV) were obtained from the dissected midguts of *O. nubilalis* and *L. decemlineata* and from the whole last instar larvae of *L. botrana*, following the differential magnesium precipitation method [64,65]. Proteins in the purified BBMV were quantified following Bradford protein assay [59] and stored at −80 °C.

### 4.6. Biotin Labelling

Trypsin activated Cry1Ia protein was biotinylated by using the protein biotinylation kit from GE Healthcare (GE Healthcare, Little Chalfont, UK), as described elsewhere [66]. The eluted fractions were quantified by NanoDrop 2000 spectrophotometer (ThermoFisher Scientific, Waltham, MA, USA), analyzed by 12% SDS-PAGE and verified by Western blot. The protein fractions were concentrated by using an Amicon Ultra-4 10K centrifugal filter device (Merck Millipore, Tullagreen, Ireland) and stored at 4 °C.

The interference of biotin in oligomerization was tested following incubation of the biotin labelled Cry1Ab with *O. nubilalis* BBMV. The detection of biotin labelled Cry1Ab oligomerization was performed following the same protocol than has been described for Cry1Ia. The results showed no influence of biotin in oligomerization (Figure S1), as had been already claimed by other authors that have used biotin labelled proteins to detect oligomers [12,20,53,54].

### 4.7. Oligomerization Assays with Sf21 Cells

The confluent monolayer growing Sf21 cells were suspended in fresh Grace’s medium without FBS. The cell concentration was measured (by using the Countess Automated Cell Counter from Invitrogen, Carlsbad, California, USA), and 100 µL of cell suspension at a concentration of 2 × 10^6^ cells/mL were seeded into 96-well plates. The oligomerization assays were performed as described by Portugal et al. [38] with slight modifications. In short, cells were incubated at 25 °C for at least 30 min. Later, 4 µg of activated toxins (biotinylated Cry1Ia or unlabeled Cry1Ab) were added to the cells (final concentration of 0.03 µg protein/µL) except in controls, which received 50 mM carbonate buffer pH 10.5. The plates were incubated for 3 h at 25 °C. After the incubation, the treated cells were collected and pelleted by centrifugation at 16,200× *g*, 4 °C for 15 min. The supernatants containing unbound proteins were kept for further analysis. The controls (proteins alone, without cells) were submitted to the same experimental conditions as treatments. After centrifugation of the controls, as there was no pellet due to the absence of cells, a dilution of supernatant (containing 200 ng of selected protein) was analyzed in the gel. The Sf21 cells in the pellet were washed once with 200 µL of 50 mM carbonate buffer pH 10.5, and recovered by centrifugation (45 min, 18,800× *g*). The final pellet was resuspended in 10 µL of buffer and heated at 50 °C for 3 min. The proteins present in the sample were separated by SDS-PAGE 10% and electrotransferred onto polyvinylidene difluoride (PVDF) Western Blotting membrane (Roche Diagnostics GmbH, Mannheim, Germany). The membrane was incubated overnight in blocking buffer (PBST; 0.1% Tween 20 in phosphate-buffered saline, supplemented with 5% skimmed milk) with gentle shaking, and washed three times with PBST, before incubation with the corresponding antibodies. Cry1Ab protein was detected with polyclonal rabbit anti-Bt Cry1Ab/1Ac (1:10,000; 60 min) from Abraxis (Warminster, PA, USA) followed by secondary antibody (1:20,000; 60 min) coupled with horseradish peroxidase (HRP) (Sigma-Aldrich, Saint Louis, MO, USA), whereas biotinylated Cry1Ia protein was detected by streptavidin-conjugated horseradish peroxidase (1:2000; 60 min) (GE Healthcare, Amersham, UK). Both Cry1Ab and Cry1Ia proteins were visualized by chemiluminescence using ECLTM prime western blotting detection reagent (GE Healthcare, Little Chalfont, UK) using an ImageQuant LAS400 image analyzer (GE Healthcare Bio-Sciences, Upsala, Sweden). The molecular weight marker used was Precision Plus Protein™ Dual Color Standard (Bio-Rad, Carlsbad, CA, USA). Each oligomerization assay was repeated at least three times. 

### 4.8. Oligomerization Assays with BBMV

The BBMV were centrifuged for 10 min at 16,000× *g* and suspended in 50 mM carbonate buffer pH 10.5. The oligomerization protocol was set up after reviewing the procedures described in the literature for Cry1 proteins [17,18,37,38,39,45,47,51,52,54]. Finally, the oligomerization assays with BBMV were performed following Ocelotl et al. [51] who employed the milder SDS-PAGE denaturing conditions (heating the samples 3 min at 50 °C), with some modifications. Briefly, 2 µg of biotin labelled activated Cry1Ia and activated Cry1Ab toxins were incubated for one hour with 5 µg of *L. botrana* or *L. decemlineata* BBMV, or with 20 µg of *O. nubilalis* BBMV, at 37 °C, in a final volume of 50 μL. Activated proteins incubated in the absence of BBMV and samples containing only BBMV were used as controls. Then, phenylmethylsulfoyl fluoride (PMSF) was added (final concentration 1 mM) and BBMV were recovered by centrifugation at 18,400× *g* for 45 min at 4 °C. The supernatant containing unbound protein was recovered and stored. The controls (samples with proteins and without BBMV) went through the same experimental conditions as treatments. After centrifugation of the controls, a dilution of the supernatant (containing 200 ng of selected protein) was analyzed in the gel to avoid the observation of the over saturated signal in the membrane. In the samples containing BBMV, the pellet was washed once with 100 μL ice-cold buffer. The final BBMV pellets were resuspended in 10 µl of the buffer. After incubating the samples for 3 min at 50 °C, the proteins were separated by 10% SDS-PAGE and blotted onto PVDF Western Blot membranes (Roche Diagnostics GmbH, Mannheim, Germany). After Western blot, Cry1Ab was detected with polyclonal rabbit anti-Bt Cry1Ab/1Ac, and Cry1Ia was detected by streptavidin-conjugated horseradish peroxidase as has been described in the previous section. The experiments were repeated, at least, three times. 

## Figures and Tables

**Table 1:** Toxicity parameters of Cry1Ab and Cry1Ia protoxins.

Insect Species	Bt Toxins	LC_50_	Fiducial Limits (95%)		Regression Line	
			Lower	Upper	Slope ± SE	a ± SE
*L. botrana*	Cry1Ab	153	106	217	1.15 ± 0.13	2.48 ± 0.3
	Cry1Ia	80	56	108	1.28 ± 0.13	2.56 ± 0.31
*O. nubilalis*	Cry1Ab	69	47	97	1.06 ± 0.11	3.04 ± 0.23
	Cry1Ia	273	88	1011	1.50 ± 0.15	1.34 ± 0.14
*L. decemlineata*	Cry1Ab	NT	-	-	-	-
	Cry1Ia	22	12	53	0.51 ± 0.07	4.29 ± 0.09

LC_50_ values are expressed as ng/cm^2^ for *L. botrana* and *O. nubilalis*, and in μg/mL for *L. decemlineata*. NT: non-toxic at 100 µg/mL.

## Supplementary Materials

The following is available online at https://www.mdpi.com/2072-6651/12/2/133/s1, Figure S1: Biotin labelled Cry1Ab oligomer formation promoted by *O. nubilalis* BBMV.
