# Supplementary material for: Study of the Bacillus thuringiensis Cry1Ia Protein Oligomerization Promoted by Midgut Brush Border Membrane Vesicles of Lepidopteran and Coleopteran Insects, or Cultured Insect Cells
Source: Toxins (Basel). 2020 Feb 21;12(2):133. doi: 10.3390/toxins12020133 (PMC7076784; doi:10.3390/toxins12020133)
Supplement: Supplementary file 1 [file toxins-12-00133-s001.pdf]

Supplementary Materials: Study of the *Bacillus thuringiensis* Cry1Ia Protein Oligomerization Promoted by Midgut Brush Border Membrane Vesicles (BBMV) of Lepidopteran and Coleopteran Insects, or Cultured Insect Cells

Ayda Khorramnejad, Mikel Domínguez-Arrizabalaga, Primitivo Caballero

Baltasar Escriche and Yolanda Bel

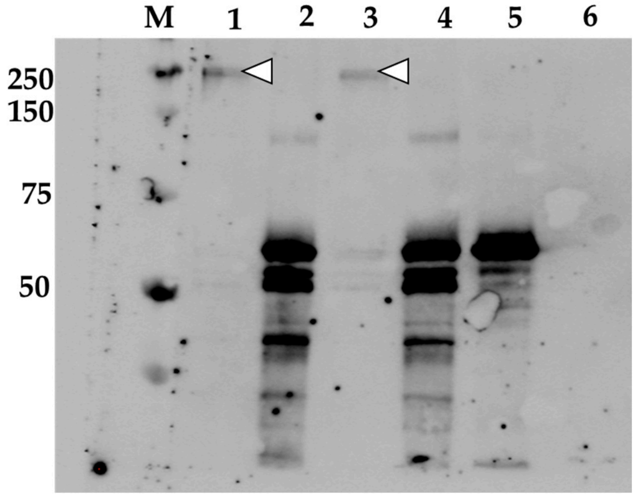

Figure S1. Biotin labelled Cry1Ab oligomer formation promoted by *O. nubilalis* BBMV. Lanes 1 and 3- Proteins associated with the BBMV (pellet) after incubation with Cry1Ab protein. Lanes 2 and 4- Supernatant after incubation of BBMV-proteins. Lane 5- Control of Cry1Ab protein incubated without BBMV. Lane 6- *O. nubilalis* BBMV. Lane M- molecular weight marker. The arrowhead points to the Cry1Ab oligomer (about 250 kDa).
